# Correction: The deubiquitinase USP45 inhibits autophagy through actin regulation by Coronin 1B

**DOI:** 10.1083/jcb.20240701403262025c

**Published:** 2025-04-04

**Authors:** Yuchieh Jay Lin, Li-Ting Huang, Po-Yuan Ke, Guang-Chao Chen

Vol. 224, No. 5 | https://doi.org/10.1083/jcb.202407014| March 11, 2025

The authors regret that, in their original article, the wrong version of Fig. 2 I was used. During the revision process, this panel was mistakenly replaced with an older version. The corrected figure is shown here. This error does not affect the conclusions of the study, and the legend of the figure remains unchanged.

In addition, the authors identified typographic errors in the Results section and in several figure legends. The corrected text is shown below.

These errors appear in print and in PDFs downloaded on or before March 26, 2025.

**Figure:**
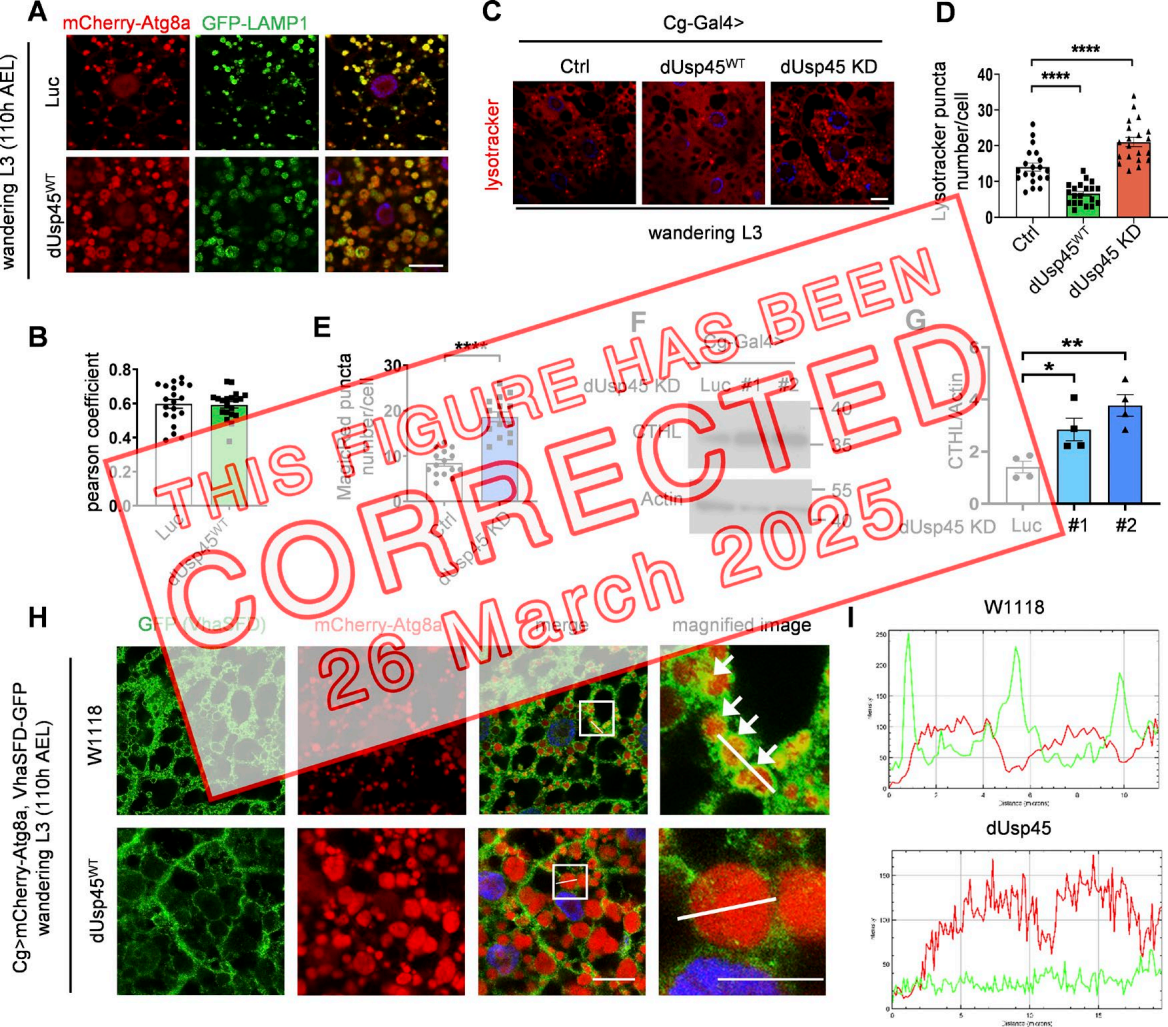


**Figure 2.**
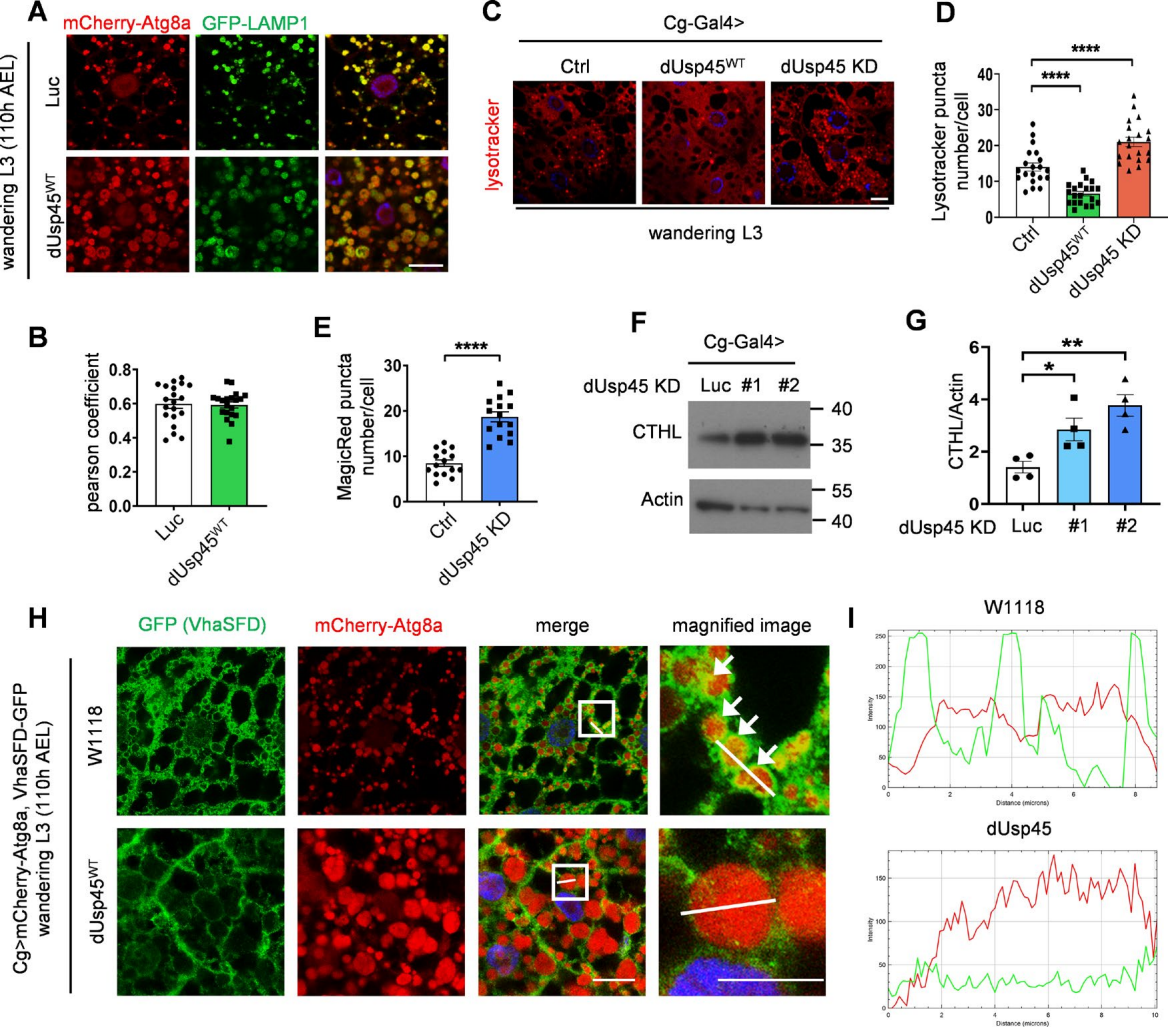
**dUsp45 overexpression impairs lysosomal acidification and V-ATPase lysosomal localization. (A)** Confocal microscopy analysis showedcolocalization of mCherry-Atg8a and GFP-LAMP1 in control or dUsp45^WT^ expressing wL3 larval fat body cells. Scale bar, 20 μm. **(B)** Quantification of the colocalization of Atg8a and LAMP1 in A. Pearson’s correlation coefficient was analyzed by ImageJ. Data are shown as mean ± SEM, *n* = 3, ≥ 20 cells. **(C)** Confocal microscopy analysis of wL3 larval fat body cells expressing *Luc* (Ctrl), *dUsp45*^*WT*^, or *dUsp45*^*RNAi*^ with *Cg-Gal4* driver and stained with the fluorescent dye LysoTracker Red. Scale bar, 20 μm. **(D)** Quantification of the number of LysoTracker-positive dots per cell in C; data shown as mean ± SEM, *n* = 3, ≥ 20 cells. **(E)** Quantification of the number of MagicRed puncta per cell in *dUsp45* knockdown wL3 larval fat body cells. Data are shown as mean ± SEM, *n* = 3, ≥ 15 cells. **(F)** Western blot analysis of Cathepsin-L (CTHL) expression levels in the larval fat bodies expressing *Luc* (Ctrl) or *dUsp45*^*RNAi*^ under the control of *Cg-Gal4*. **(G)** Quantification of Cathepsin-L expression normalized to Actin in F. Data shown as mean ± SEM of four independent experiments. **(H)** Confocal microscopy analysis of localization of VhaSFD (GFP positive) and mCherry-Atg8a in control and dUsp45WT overexpression wL3 larval fat body cells. The arrows showed the colocalized signals of VhaSFD and Atg8a. The scale bars showed 20 μm (original) and 10 μm (zoom-in). **(I)** Line-scan profiles of fluorescence intensity for mCherry-Atg8a and GFP-VhaSFD along the white line in H. Significance was determined using one-way ANOVA and Dunnett’s multiple comparisons test (D and G), and Student’s *t* test (B and E); *P < 0.05; **P < 0.01; ****P < 0.0001. Source data are available for this figure: SourceData F2.

In the Results section, under “Loss of dUsp45 extends lifespan and ameliorates polyQ-induced eye degeneration in *Drosophila*,” the following text was changed:

To further determine the role of dUsp45 in aging and longevity, we examined the lifespan of dUsp45 mutant flies, including the dUsp45 homozygote P-element insertion mutant (dUsp45^EP1400^/dUsp45^EP1400^dUsp45^EY19835^/dUsp45^EY19835^) and the dUsp45 transheterozygote mutant (dUsp45^EP1400^dUsp45^EY19835^/dUsp45B) (Fig. 8 E).

Figure 3. Mammalian USP45 negatively regulates autophagy.

(C) Quantification of the number of LC3 puncta in control and USP45 knockdown cells in A**B**; data shown as mean ± SEM, *n* = 3, ≥ 30 cells.

Figure 4. USP45 negatively regulates lysosomal activity by altering V-ATPase endolysosomal trafficking.

(B) Quantification of mature CTHL expression normalized to tubulin in HA.
(D) Quantification of the number of DQ-BSA puncta in control and USP45 knockdown cells treated as J**C**; data are shown as mean ± SEM, *n* = 3, ≥ 30 cells.

Figure 6. USP45 depletion–induced F-actin patch formation is required for V-ATPase translocation to lysosomes. (A) Confocal microscopy analysis of F-actin structures stained with phalloidin in control (scr) and *USP45* knockdown HeLa cells. Arrows indicate cytoplasmic F-actin patches. Scale bar, 20 μm. (B) Quantification of the area of cytoplasmic F-actin patches in A; data shown as mean ± SEM, *n* = 3, ≥ 35 cells. (C) Immunofluorescence analysis of co-localization of the F-actin patches and endolysosomal markers (EEA1, Rab7, and LAMP2**LAMP1**) in control and USP45 knockdown cells with indicated antibodies. The scale bars showed 20 μm (original) and 5 μm (zoom-in)**insets with 1.5x zoom**. (D) Quantification of the colocalization of F-actin patches and endolysosomal markers in C. Pearson’s correlation coefficient was analyzed by ImageJ. Data are shown as mean ± SEM, *n* = 3, ≥30 cells. (E) Immunofluorescence analysis of colocalization of V-ATPase (V1H) and lysosome (LAMP1) in control and *USP45* knockdown HeLa cells treated with LatA (200 nM) or Arp2/3 inhibitor CK666 (200 μM) for 2 h. Scale bar, 10 μm (original) and 5 μm (zoom-in)**insets, 2.5x zoom**. **(F)** Quantification of the colocalization of V1H and LAMP1 in E. Pearson’s correlation coefficient and colocalization rate were analyzed by ImageJ. Data are shown as mean ± SEM, *n* = 3, ≥ 25 cells. (G) Immunofluorescence analysis of colocalization of V-ATPase (V1H) and lysosome (LAMP1) in control and *USP45* knockdown cells transfected with control (siCtrl) siRNA or siRNA targeting WASP family genes (siN-WASP and siWASH). Scale bar, 10 μm (original) and 2.5 μm (zoom-in)**insets, 2.5x zoom**. (H) Quantification of the colocalization of V1H and LAMP1 in G. Data are shown as mean ± SEM, *n* = 3, ≥ 25 cells. Significance was determined using one-way ANOVA and Dunnett’s (B) or Tukey’s (F and H) multiple comparisons test, and Student’s *t* test (D); ***P < 0.001; ****P < 0.0001; NS not significant.

Figure 7. Depletion of Coro1B promotes autophagy and V-ATPase lysosomal localization. (A) Confocal images of late L2 larval fat body cells expressing GFP-mCherry-Atg8a in control (Ctrl) and Coro knockdown larvae. Scale bar, 20 μm. (B) Quantification of the ratio of autolysosomes (GFP^−^ mCherry^+^) to total Atg8a puncta per cell in A; data are shown as mean ± S EM, *n* = 3, ≥20 cells. (C) Confocal images of wL3 larval fat body cells from flies expressing mCherry-Atg8a and indicated transgenes driven by *Cg-Gal4*. Scale bar, 20 μm. (D) Quantification of Atg8a puncta number and size shown in C. Data are shown as mean ± SEM, *n* = 3, ≥ 15 cells. (E) Immunofluorescence analysis of colocalization of V-ATPase (V1H) and lysosome (LAMP1) in control and *Coro1B* knockdown HeLa cells. Scale bar, 20 μm (original) and 2.5 μm (zoom-in)**insets, 3x zoom**. (F) Quantification of the colocalization of V1H and LAMP1 in E. Pearson’s correlation coefficient and colocalization rate were analyzed by ImageJ. Data are shown as mean ± SEM, *n* = 3, ≥ 20 cells. (G) Immunofluorescence analysis of colocalization of V-ATPase (V1H) and lysosome (LAMP1) in control and *Coro1B* knockdown cells treated with LatA (200 nM) or Arp2/3 inhibitor CK666 (200 μM) for 2 h. Scale bar, 20 μm (original) and 5 μm (zoom-in)**insets, 2x zoom**. (H) Quantification of the colocalization of V1H and LAMP1 in G. Data are shown as mean ± SEM, *n* = 3, ≥ 25 cells. Significance was determined using one-way ANOVA and Tukey’s multiple comparisons test (D and H), and Student’s *t* test (B and F); ****P < 0.0001; NS not significant.

Figure 8. Loss of dUsp45 extends lifespan and mitigates polyQ-induced toxicity in *Drosophila*.

(B) Quantification of CTHL levels normalized to Tubulin in A. Data are shown as mean ± SEM of four**three** independent experiments.
(D) Quantification of dUsp45 mRNA levels normalized to Actin. Data are shown as mean ± SEM of four**three** independent experiments.

Figure S1. dUsp45 impairs autophagy and V-ATPase translocation to the autolysosome.

(G) Quantification of autolysosome size in D**F**; data shown as mean ± SEM, *n* = 3, ≥ 20 autolysosomes.

Figure S3. USP45 interacts with Coro1B and mediates its ubiquitination. (A) Immunoprecipitation analysis of interaction between Flag-Coro1B and Myc-USP45 bywith Flag antibody. (B) Immunoprecipitation analysis of the interaction between FlagCoro1B and endogenous USP45 bywith pulldown of Flag-Coro1B. (B)(C) RT-PCR analysis of CORO1B mRNA level in control and USP45 knockdown HeLa cell. Quantification of CORO1B mRNA level was normalized to GAPDH. Data are shown as mean ± SEM from three independent experiments. (C)(D) Immunoprecipitation analysis for Flag-Coro1B ubiquitination in HEK293T cells with expression of wild-type (WT) or mutant (C199A) MycUSP45. Cells were lysed by a denaturing agent (1% SDS) containing buffer. Quantification of Ub was normalized to Flag-Coro1B. Data are shown as mean ± SEM from three independent experiments. (D) Significance was determined using one-way ANOVA and Dunnett’s multiple comparisons test (C and D); ****P < 0.0001. Source data are available for this figure: SourceData FS3.

