# Supplementary figures and images for: The deubiquitinase USP45 inhibits autophagy through actin regulation by Coronin 1B
Source: J Cell Biol. 2025 Mar 11;224(5):e202407014. doi: 10.1083/jcb.202407014 (PMC11895698; doi:10.1083/jcb.202407014)

**Fig1F**

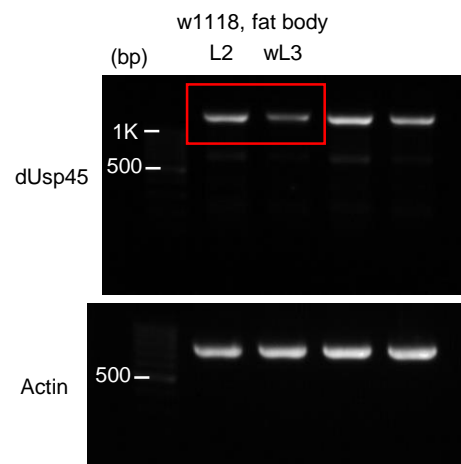

Supplement: SourceData F1 — is the source file for Fig. 1. [file jcb_202407014_sourcedataf1.pdf]

**Fig 2F**

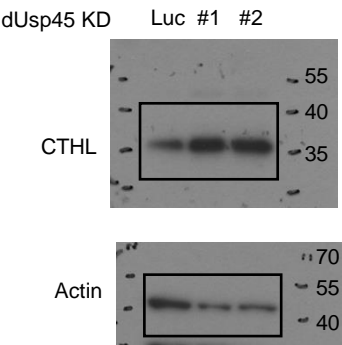

Supplement: SourceData F2 — is the source file for Fig. 2. [file jcb_202407014_sourcedataf2.pdf]

**Fig. 3D**

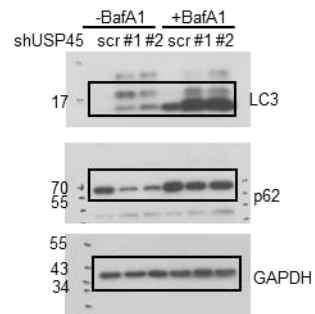

**Fig. 3H**

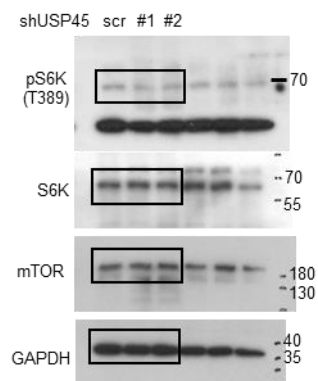

**Fig. 3J**

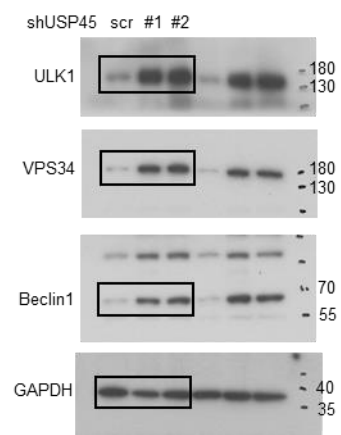

Supplement: SourceData F3 — is the source file for Fig. 3. [file jcb_202407014_sourcedataf3.pdf]

**Fig. 4A**

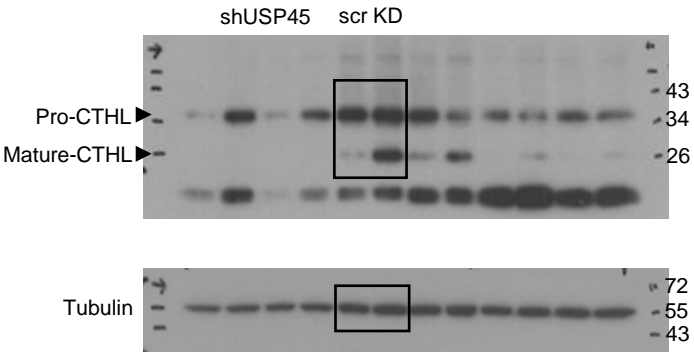

Supplement: SourceData F4 — is the source file for Fig. 4. [file jcb_202407014_sourcedataf4.pdf]

**Fig. 5A**

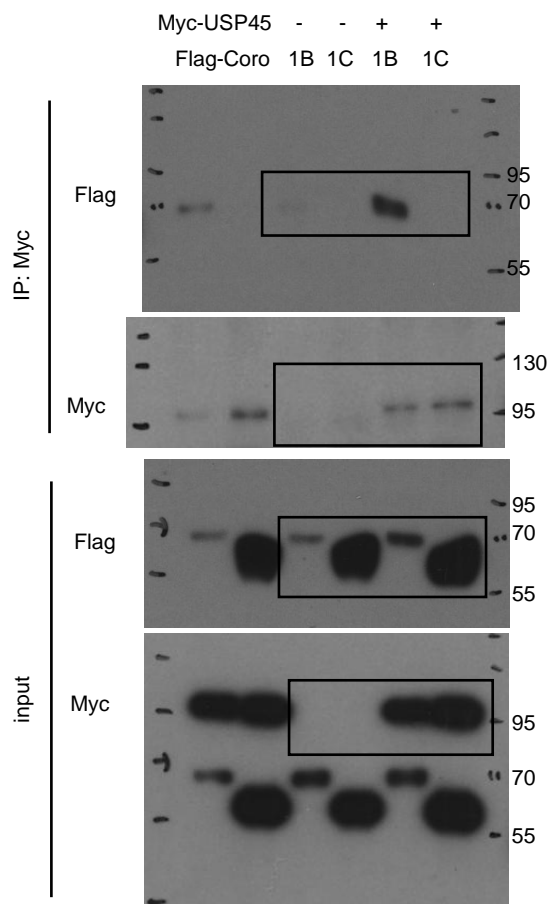

**Fig. 5B**

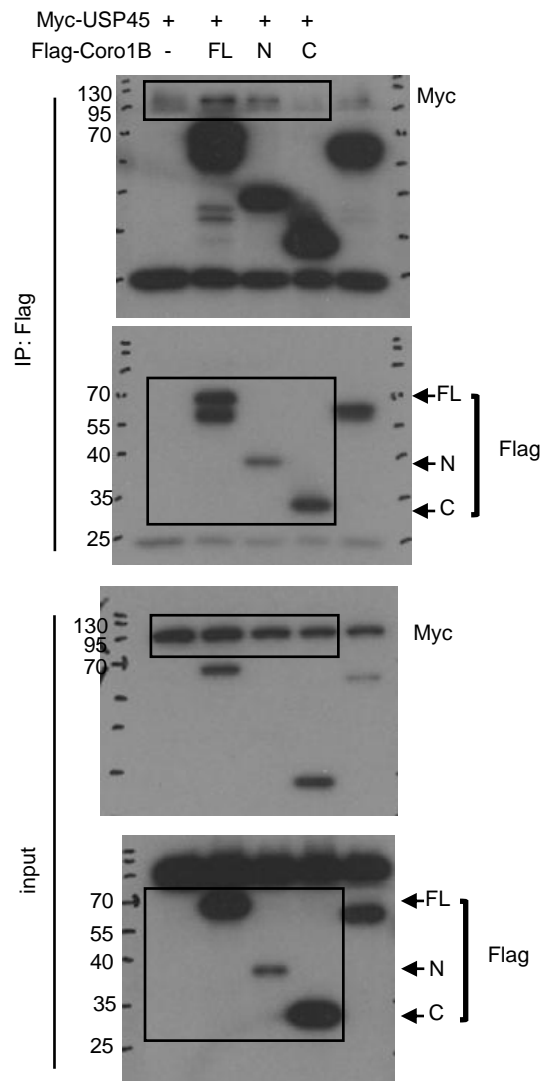

**Fig. 5C**

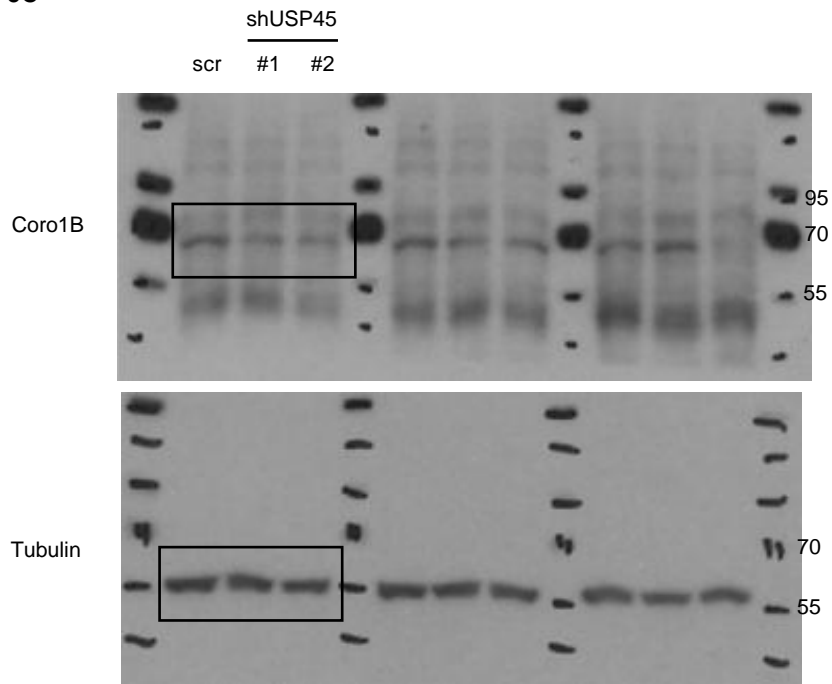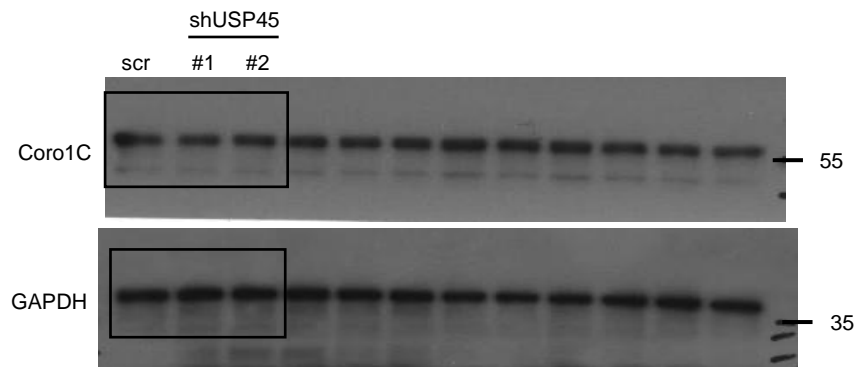

**Fig. 5E**

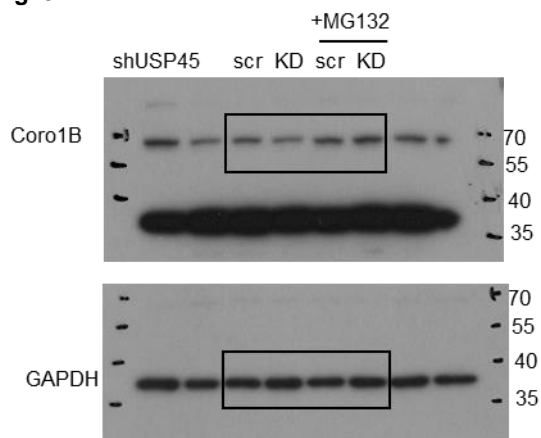

**Fig. 5G**

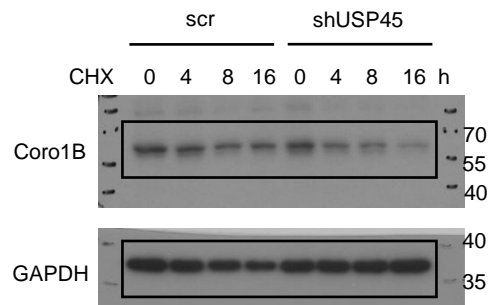

**Fig. 5I**

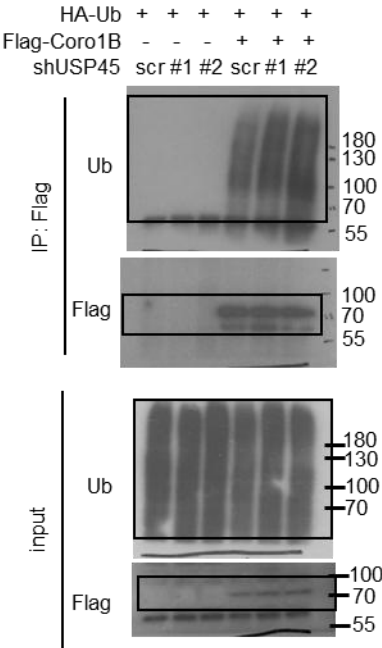

**Fig. 5K**

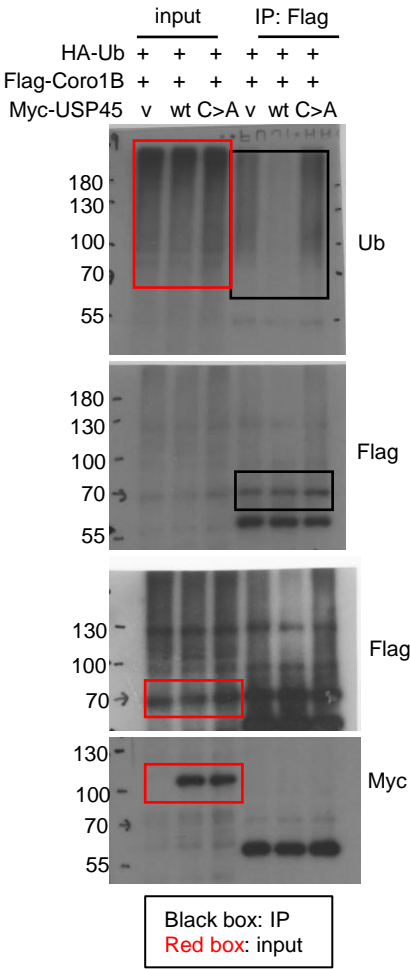

Supplement: SourceData F5 — is the source file for Fig. 5. [file jcb_202407014_sourcedataf5.pdf]

**Fig. 8A**

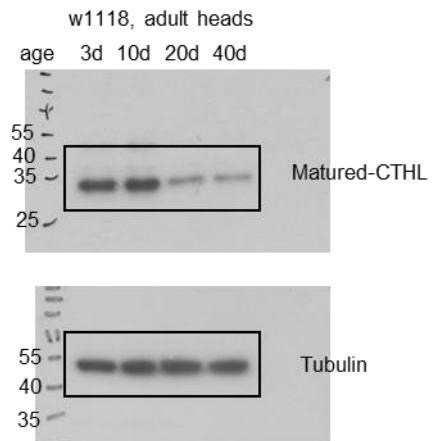

**Fig8C**

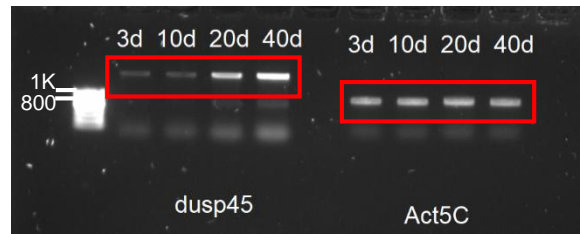

**Fig8E**

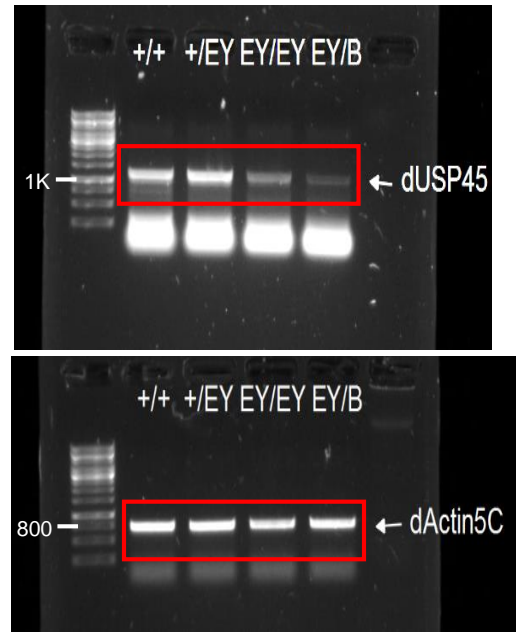

Supplement: SourceData F8 — is the source file for Fig. 8. [file jcb_202407014_sourcedataf8.pdf]

**Fig. S1E**

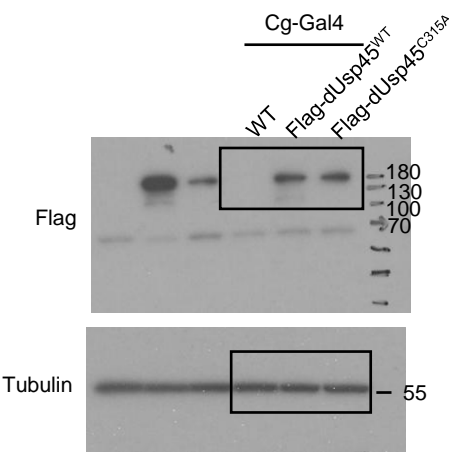

**Fig. S1H**

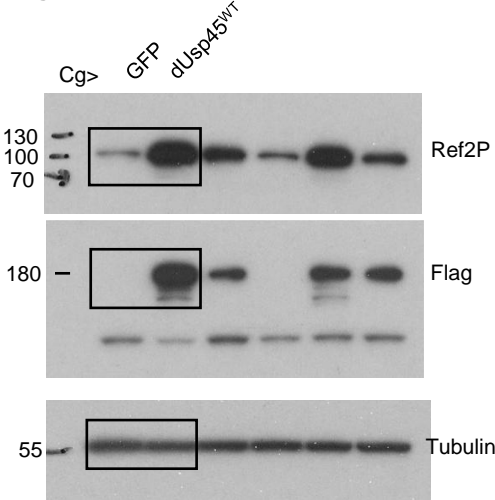

Supplement: SourceData FS1 — is the source file for Fig. S1. [file jcb_202407014_sourcedatafs1.pdf]

Fig. S2C

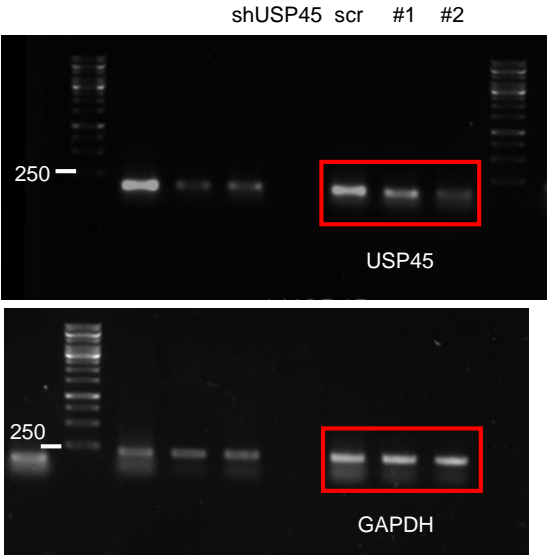

Fig. S2H

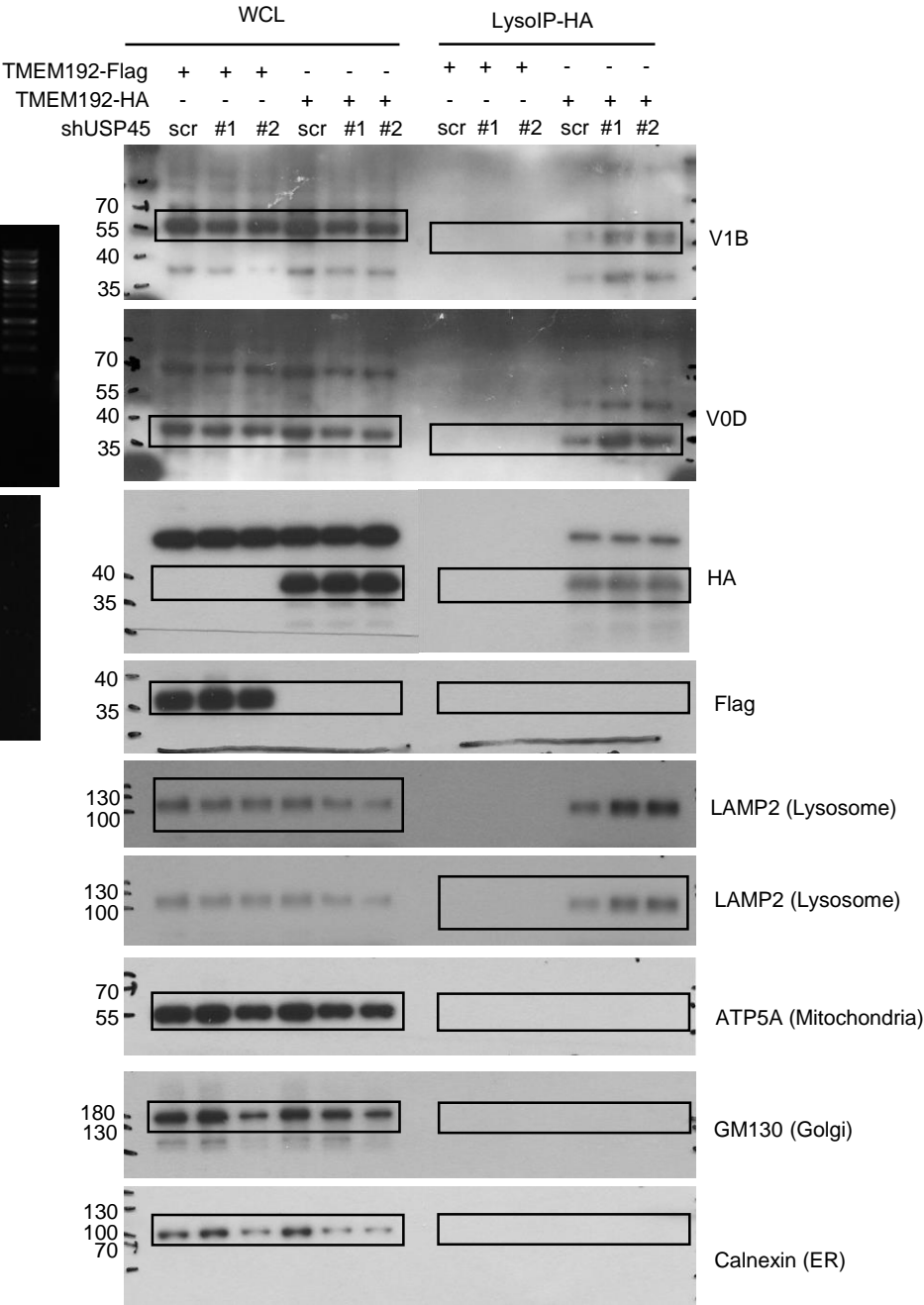

Supplement: SourceData FS2 — is the source file for Fig. S2. [file jcb_202407014_sourcedatafs2.pdf]

**Fig. S3A**

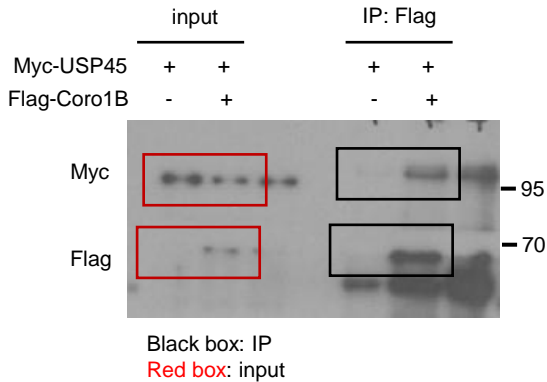

**Fig. S3B**

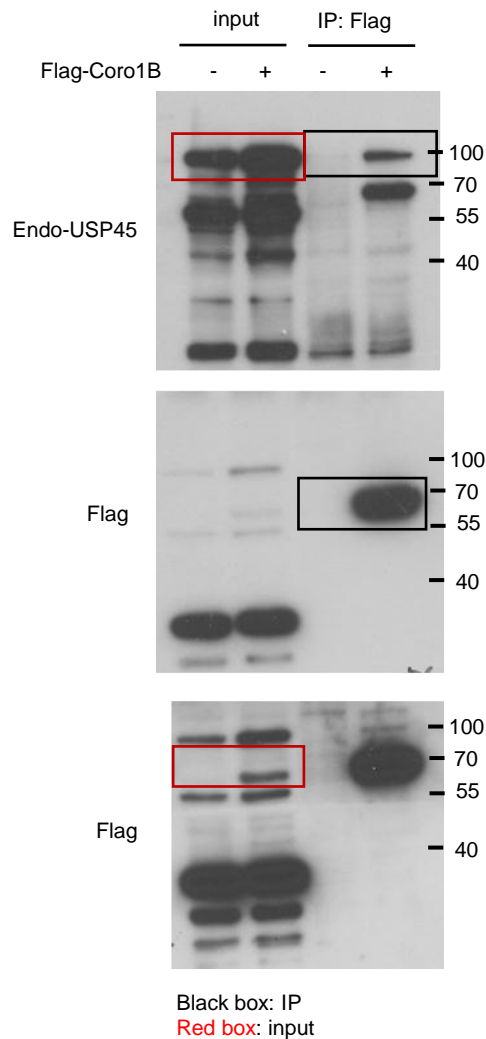

Fig. S3C

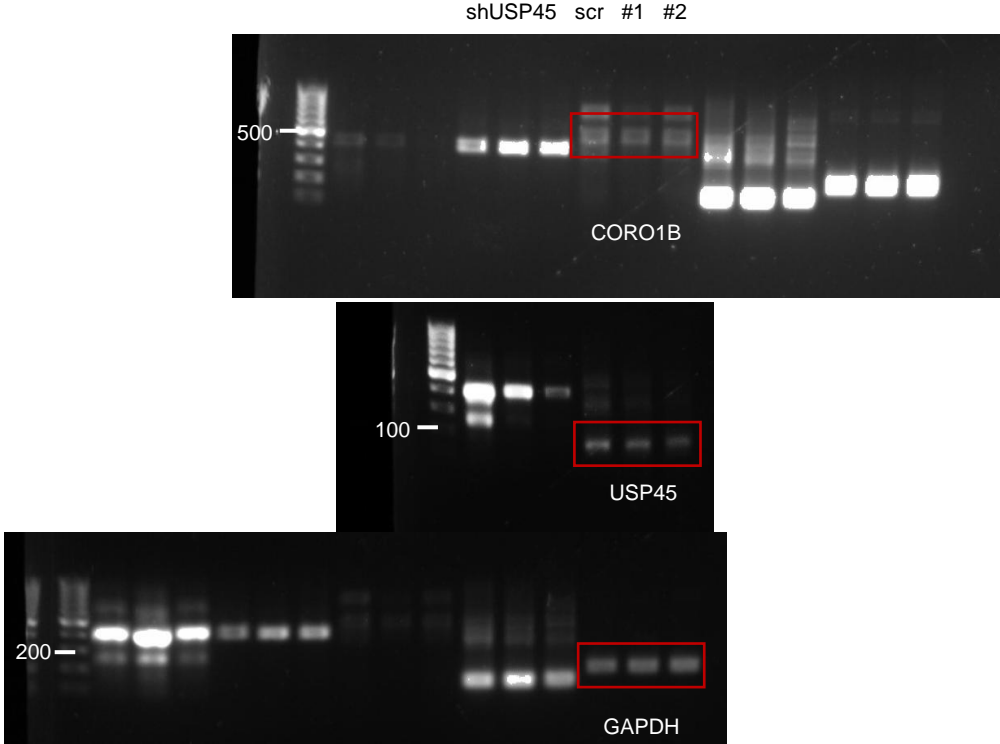

Fig. S3D

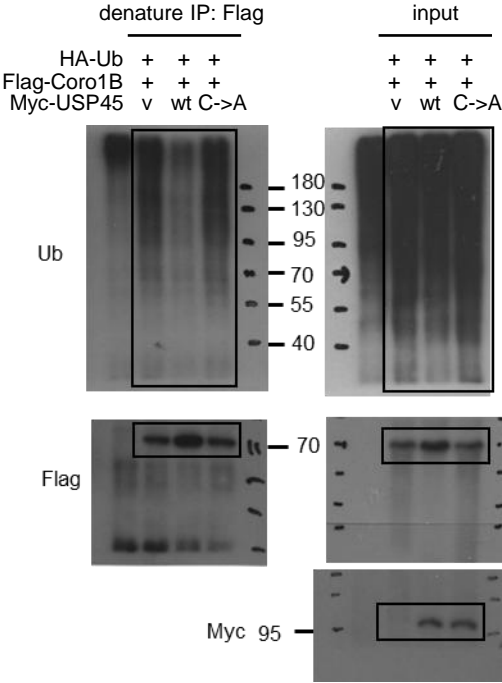

Supplement: SourceData FS3 — is the source file for Fig. S3. [file jcb_202407014_sourcedatafs3.pdf]

Fig. S5G

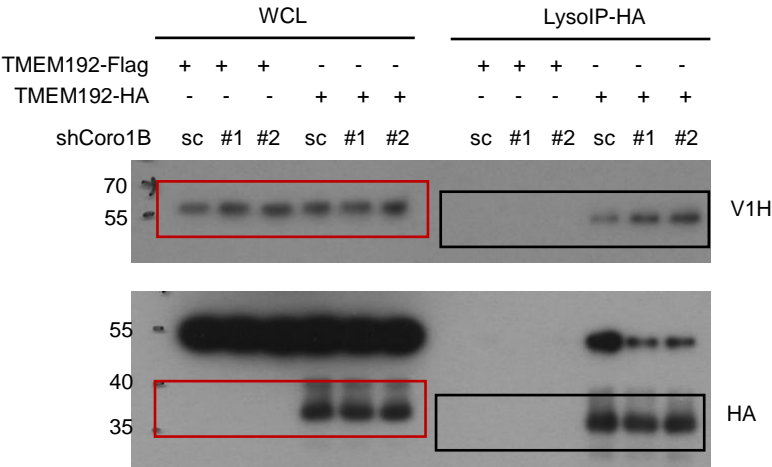

Supplement: SourceData FS5 — is the source file for Fig. S5. [file jcb_202407014_sourcedatafs5.pdf]
